# An experimental workflow to assess the applicability of microemulsions for conformance improvement in oil-bearing reservoir

**DOI:** 10.1016/j.heliyon.2023.e17667

**Published:** 2023-06-27

**Authors:** Nilanjan Pal, Yara Alzahid, Abdulkareem M. AlSofi, Muhammad Ali, Nurudeen Yekeen, Hussein Hoteit

**Affiliations:** a Ali I. Al-Naimi Petroleum Engineering Research Center (ANPERC), Physical Science & Engineering Division, King Abdullah University of Science and Technology, Thuwal, Saudi Arabia; b Department of Petroleum Engineering & Earth Sciences, Indian Institute of Petroleum and Energy (IIPE), Visakhapatnam, India; c EXPEC Advanced Research Center, Saudi Aramco, Dhahran, Saudi Arabia; d Department of Chemical and Petroleum Engineering, UCSI University, Kuala Lumpur 560000, Malaysia

**Keywords:** Microemulsion, Phase behavior, Salinity effect, Stability, Viscosity, Conformance improvement, Workflow design

## Abstract

A comprehensive workflow approach is necessary to link multiple experimental tasks and identify microemulsion (ME) formulations with ‘optimal’ stability, displacement behavior and technical feasibility in the petroleum industry. In this paper, a systematic approach is described with the aid of a case study which involves the formulation of an anionic sodium dodecyl sulfate-based microemulsion. The design of such ME systems requires a proper methodology, substantial laboratory work, and functional assessment from research/industrial viewpoints. The surfactant has been screened in terms of its micellization potential, followed by phase behavior analysis and Winsor classification of prepared microemulsions. The desired composition(s) are characterized via several tools to determine droplet size, morphology, oil/water solubilization potentials and salinity scan results. The suitability of the microemulsion system for conformance improvement technology (CIT) is proposed to be assessed via physicochemical evaluation studies encompassing two attributes: rheology and stability. For a favorable ‘conforming’ drive, the microemulsion must exhibit phase stability, sufficient injectivity, and moderate-to-high viscosity under shear. Technical assessment by the industry and research team must also include factors related to cost, availability of chemicals, environmental degradation, and reservoir considerations. The article demonstrates a comprehensive all-inclusive workflow methodology to design and formulate surfactant-stabilized microemulsions via case study analysis for application in CIT. This represents a sound approach to identifying efficient, cost-effective injection fluid systems and provides a framework to identify useful parameters for ME formulation design and employ the proposed (effective) strategy for conformance control.

## Graphical abstract

**Figure:**
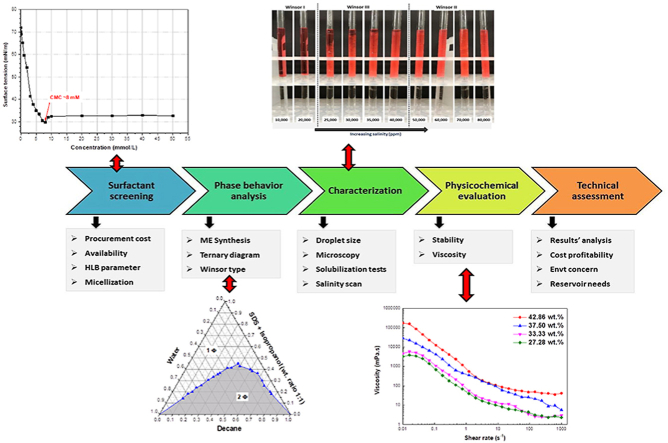

## 1 Introduction

In current scenario, the need to extend the production life of mature hydrocarbon reservoirs is a major challenge for the oil industry. Problems related to excess water production pose a huge concern in depleting reservoirs, which needs to be minimized by the application of conformance improvement technology (CIT). Conventional routes such as polymer flooding, gel treatment, polymer enhanced foams (PEFs), hydraulic fracturing, and acid stimulation have shown promising conformance potential in the past few years. However, these techniques are associated with the persistence of instability, degradability and economic issues during operation [[1], [2], [3], [4]]. Recently, a relatively novel chemical system i.e. microemulsion (ME) has gained momentum as a potential alternative for well conformance owing to desirable structural and flow behavioral attributes. Winsor [5] further categorized the different kinds of ME in terms of their varying phase behavior and micellar structure/arrangement. Microemulsions, first termed by Schulman [6], refer to optically isotropic dispersions of oil/water which consist of an amphiphilic component. They can be differentiated from coarse emulsions in terms of their thermodynamic stability and nanometric droplet structure [[7], [8], [9]]. Winsor I microemulsions are biphasic systems consisting of oil droplets (normal or direct micelles) dispersed in the continuous aqueous phase and are broadly categorized as oil-in-water systems. On the contrary, Winsor II systems is also a biphasic system, comprising of reverse or inverted micelles with dispersions of water in a continuous oleic phase. Winsor III corresponds as a ‘bi-continuous’ phase in equilibrium, stabilized with excess of water/oil phases in the presence of both normal and reverse micelles. Winsor IV represents a single phase, homogeneous microemulsion system that is formed at exceedingly high surfactant dosages and, hence, uneconomical in application. Though the description of microemulsions has undergone several alterations in the past few decades, their functionality has only increased in numerous applications including catalytic reactions, food delivery, cosmetics, pharmaceuticals, drug delivery, and petroleum sector [[10], [11], [12], [13], [14]]. Studies concerning the application of microemulsions for conformance control is not extensive. This is primarily due to the lack of technical know-how and injection strategy in petroleum formations. The current study delves into these gaps in research investigations, and integrates experimental results via case study analysis to develop a comprehensive laboratory workflow.

The successful application of microemulsion-assisted conformance improvement technology (ME-CIT) lies in the attainment of two primary traits: stability and viscosity [[15], [16], [17]]. Lin et al. [18] proposed that ME stability is a function of inter-droplet coalescence and the nature of surfactant-stabilized nuclei. For ionic surfactant systems, kinetic stability is determined is predicted in terms of the electrical double layer repulsion whereas the stabilization phenomenon is debated for non-ionic surfactant MEs [19]. However, an additional effect known as steric stabilization also aids in improving the integrity of formed droplets in the ME phase. The viscosity of microemulsions affects the fluid mobility ratio during porous media flow and is an important conformance parameter. It must be configured to ensure invasion of conformance fluid to the specified reservoir zone of interest as well as preserve injectivity [4]. Acid microemulsions increase the oil recovery efficiency of the reservoir, but may pose detrimental effects for water production control [20,21]. Romero [22] observed that emulsion viscosity is dependent on the capillary number and suggested its potential as a mobility control agent. Ni and others [16] developed a simulation model to corroborate the effect of viscosity on fluid diversion characteristics of emulsion. The scope of ME fluids has broadened owing to their functional dexterity and gradual shift in industry requirements from enhanced oil recovery application in the past to conformance improvement technology (CIT).

A detailed, efficient workflow is required for the optimization of favorable ME formulations from a laboratory approach. Surfactant screening via phase behavior tests is necessary to design formulations that exhibit sufficient solubilization and the ability to plug high permeability regions in a specific reservoir zone under dynamic shear conditions [4]. The stability and rheology of microemulsions provide a functional approach to assess its behavior in oil-rock-water interfaces [[23], [24], [25]]. Budhathoki et al. [26] conducted tests for surfactant-based ME and confirmed their favorable stability, robust nature and low coalescence rate over a period of about 30 min. A sufficiently viscous ME is capable of blocking pore channels associated with high water production and subsequently improves the mobility ratio through low permeability zones [4,17]. To date, several studies have focused on the design and optimization of ME for application in oil recovery [12,[27], [28], [29]]. Nevertheless, little consideration has been given to the design of a proper all-inclusive experimental workflow that aims to predict the suitability of a specific {oil-surfactant-cosurfactant-water} based microemulsion for CIT. If the formulation strategy of ME are regulated and optimized properly on a laboratory scale, the area of microemulsion-assisted conformance control can prove to be an effective tool with promising repercussions for the petroleum industry. The current study delves into these gaps in research investigations, and integrates experimental results via case study analysis to develop a comprehensive laboratory workflow.

The present article describes a comprehensive formulation methodology for the ME-CIT process, including a step-by-step process from initial surfactant evaluation till microemulsion formulation. This has been conducted by introducing a specific (anionic surfactant) case study to prepare, formulate and optimize microemulsion composition for suiting the reservoir’s conformance needs.

## 2 Towards a comprehensive laboratory workflow: a case study analysis

**Fig. 1:**
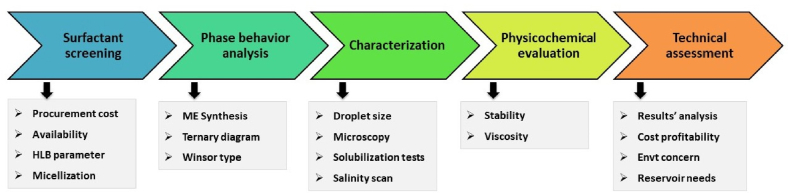
Proposed laboratory workflow of microemulsion formulation for application in conformance improvement technology (CIT).

The article focusses on presenting a comprehensive technical workflow to formulate and assess the viability of surfactant-based microemulsions for application in CIT. In earlier works of research, most ME fluid systems were investigated and employed by the petroleum sector for enhanced oil recovery applications [[29], [30], [31], [32]]. In that scenario, ultra-low interfacial tension and wettability alteration represent primary characteristics to attain favorable oil displacement through rock pores [29,30,32]. However, conformance treatment primarily aims to reduce water production by plugging high permeability pore channels [17,33,34]. Two primary requirements of a ME conformance fluid has been recognized as a result of this investigation, i.e. stability and viscosity enhancement. Fig. 1 illustrates a workflow design to identify optimal, effective ME formulation(s) for application in CIT.

A step-by-step workflow for ME formulation methodology is suggested based on case study analysis for an anionic sodium dodecyl sulfate (SDS) surfactant in the laboratory. The conformance methodology includes five matrix avenues: surfactant evaluation, ME phase behavior analysis, characterization, physicochemical analysis, and technical results assessment. In a broader sense, the laboratory workflow begins with surfactant procurement and evaluation for initial testing. Surfactant compound for ME preparation is selected based on cost, availability, hydrophilic-lipophilic balance (HLB), and micellization behavior. Thereafter, ME is prepared by stirring under certain parameters, and its phases are investigated to identify Winsor type categories for different compositions. Once suitable Winsor 2+ phase(s) have been identified for appropriate function, i.e. conformance control, the ME compositions are characterized by one or more tools in the laboratory. These comprise dynamic light scattering tests, microscopy, solubilization studies, and/or salinity scan. This optimizes/controls the ME composition to a particular concentration (dosage) range. The optimal ME formulations are analyzed in terms of two important properties, namely, stability and dynamic viscosity behavior. It may not be necessary to employ all studies related to ME characterization, but physicochemical evaluation must be conducted in terms of stability and viscosity via research. There are factors concerning the integrity and functionality of injected MEs during reservoir conformance improvement. However, several reported that the results must be valid since they observed similar observations for pilot-testing and multiphase flow experiments in the laboratory [24,35,36]. Finally, the entire testing regime is assessed with physical, chemical and economic considerations in a holistic manner.

## 3 Materials and methodology

### 3.1 Materials required

Decane, employed as oil phase, was obtained from Sigma Aldrich Company. The co-surfactant, isopropanol (propan-2-ol), was procured from Fischer Chemicals. Distillation apparatus in the laboratory was used to obtain the de-ionized (DI) water for the aqueous phase. Sodium chloride, NaCl (99% purity, Sigma Aldrich analytical grade) add to the salinity of aqueous/microemulsion systems. Anionic surfactant, sodium dodecyl sulfate (SDS) was purchased from Sigma Aldrich Company.

### 3.2 Microemulsion preparation

Microemulsions were prepared via homogeneous mixing of components at 298 K. For the process, heptane, sodium dodecyl sulfate (SDS), isopropanol and water were used as oleic phase, surfactant, co-surfactant and aqueous phases respectively. For the construction of ternary phase diagram, the component compositions were varied. However, for ME characterization tests, the oil and water volumes were kept equal (1:1 by volume). Surfactant and co-surfactant were introduced into the solution in a 1:1 ratio (by weight). The phase behavior/equilibrium state was verified by visual observation, wherein the different phases were identified up to a specified period. Microemulsions were formed spontaneously in the presence of SDS and isopropanol, when the oil and water were brought into contact and stirred with a magnetic stirrer. Surfactant acts as emulsifier, and reduces the oil-water interfacial tension (IFT). The addition of co-surfactant (isopropanol) molecules improved the stability of the microemulsion phase stabilized by anionic surfactant micelles.

### 3.3 Phase diagram construction

For ternary phase diagram experiments, synthetic emulsions were prepared with stepwise addition of components followed by mixing and phase classification. The cosurfactant-to-surfactant (c/s) ratio was incorporated as 1:1 by weight during phase behavior analyses. Phase behavior investigations demonstrate a viable approach to determine the oil, water, and emulsifier concentrations with which stable microemulsions may be formed.

### 3.4 Microemulsion characterization

The microemulsion system has been initially characterized in terms of droplet size behavior using dynamic light scattering (DLS) tests at 298 K using Malvern Nano-ZS ZEN 3600 instrument. The absorbance of ME samples was found to be 2.33 and their refractive indices were measured as 1.43 with a portable refractometer. In this method, the oil-water ratio is kept constant at 1:1 whilst varying surfactant/cosurfactant concentration to identify the average micelle size of the sample in a quartz cuvette.

The solubilization parameter is an important parameter to validate the nature and formulation of MEs for petroleum research applications. Solubilization parameters have been determined from the volumes of oil and water solubilized in the equilibrated microemulsion phase [12]. Test tubes containing specified ME composition were prepared in the 10,000–80,000 ppm total dissolved salts (TDS) range and then rotated. The oil solubilization (SP_o_) and water solubilization parameters (SP_w_) were determined as the ratio of the volume of oil/water solubilized in the middle-phase microemulsion phase, as shown in Equations (1), (2):

(1) $${SP}_{o}=V_{os}/V_{s}$$

(2) $${SP}_{w}=V_{ws}/V_{s}$$

In this equation, V_s_ is the volume of surfactant in the middle phase microemulsion, V_os_ is the solubilized oil volume and V_ws_ is the solubilized volume of water within the ME phase. The points of intersection, if any, of the plots describe the optimal solubilization parameter as well as the optimal salinity. The optimal salinity represents the condition, wherein the Chun-Huh interfacial tension (IFT) may be expected to be lowest and microemulsion attributes are considered to be most stable/optimum for porous media applications.

Salinity scan tests were performed to identify the Winsor type phase behaviors of microemulsions containing decane oil, SDS surfactant, isopropanol co-surfactant and brine. The ME fluids were prepared at desired compositions with a 1:1 oil-to-water ratio and 1:1 isopropanol-to-SDS ratio (by weight; 40 mM SDS). After proper mixing and equilibration, the phase behaviors were observed to identify the nature of Winsor phase behavior.

### 3.5 Physicochemical evaluation

Microemulsion stability was quantified as a function of viscosity versus time plots at 298 K. The influence of oil composition on viscosity versus shear rate plots exhibited the nature of dynamic flow behavior in the 0.01–1000 s^−1^ range. Viscosity measurements were performed using cup-and-bob (coaxial cylinder) geometry in Anton Paar MCR 102 Rheometer, in which a bob was inserted into a cylindrical cup containing desired ME fluid, maintaining a vertical gap of 1.0 μm. The drag force created by ME fluid on the inner cup surface represents the viscosity at specified shear rates. All experimental measurements were repeated at least twice to confirm the reproducibility of the results.

## 4 Conformance methodology for surfactant-assisted microemulsions

A case study highlighting detailed aspects of sodium dodecyl sulfate (SDS) surfactant has been investigated from a step-by-step experimental viewpoint. This article aims to provide an inclusive, generalized workflow and methodological framework that can be deployed by research groups engaged in the field of ME-CIT.

### 4.1 Surfactant screening: first step

**Fig. 2:**
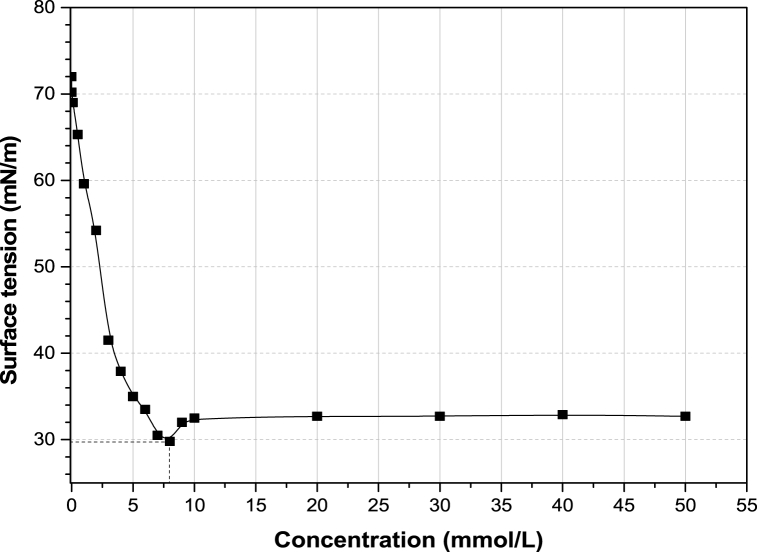
Surface tension versus concentration plots for sodium dodecyl sulfate (SDS) surfactant.

SDS is a classical surfactant consisting of robust hydrophilic and hydrophobic groups, connected by a chemical bond. It has an HLB value of 40, which suggests enhanced hydrophilicity and weak oil-solubilizing power. However, the surfactant system cannot be ignored at this stage since the ME system is a complex fluid with scope for the influence of co-surfactant addition and altered micelle chemistry. From surface tensiometry, it is evident that the anionic surfactant has a critical micelle concentration (CMC) of 8 mM, as shown in Fig. 2. The surface tension data decreased up to this limit (CMC), beyond which it increased slightly and then remained nearly constant. The surfactant molecules adsorb onto the vacant sites available at the air-water interface, till the CMC is reached [37]. Thereafter, no further accommodation sites are available for SDS molecules, resulting in a slight desorption phenomenon and an increase in surface tension. Ultimately, the interfacial system reaches a state of equilibrium and the surface tension, at this stage, is nearly constant.

### 4.2 Ternary phase diagram analysis: second step

**Fig. 3:**
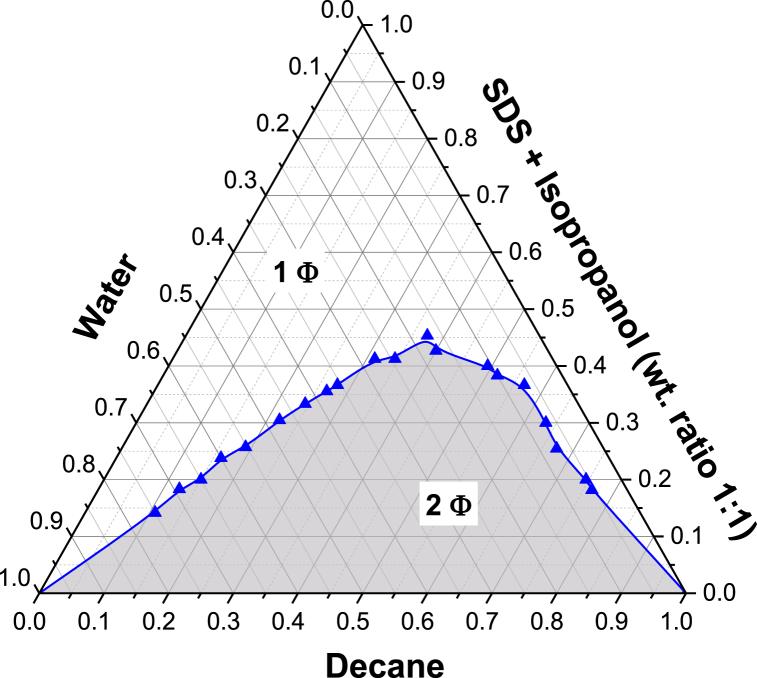
Ternary phase diagram of {decane/SDS/isopropanol/water} system with a cosurfactant-to-surfactant ratio of 1:1 at 298 K. The binodal curve (blue) separates the one-phase ME and the two-phase ME region.

One-phase and two-phase regions were identified in the pseudo-ternary phase diagrams for decane-SDS-isopropanol-aqueous systems described in Fig. 3. As shown, the ternary phase diagram of SDS microemulsion manifests a two+ (2+) phase region at low surfactant concentration initially, which gradually transforms into a large one-phase region as SDS concentration increases further (>45.33%). The one-phase or Winsor IV microemulsion exhibits favorable structure/morphology but is formed at exceedingly high surfactant concentrations, resulting in a high quantity of surfactant required and cost inefficacy [12,38]. It is indeed possible to attain single phase microemulsions with low concentration of SDS/isopropanol as emulsifier, but necessary conditions for such systems are excessively high volume of water and/or low volume of oil (might be detrimental). The 2+ region represents microemulsion systems with Winsor I, II and III phases. Each of these phases exhibits an economical approach for application in CIT due to the ability to retain a stable phase separation with either or both oil/aqueous phases [31,39]. This enhances their potential to attain an adaptive, robust flow that reaches and plugs the desired high permeability regions without too much mixing as well as retains its viscosity sufficiently under dynamic conditions. It must be noted that the dividing line in the ternary diagram is binodal, which depicts the existence of either of the two physical states, i.e. between one-phase and 2+ phase regions [40]. For CIT application, the desirability of the microemulsion is dependent on its capability to first reach and then plug specific reservoir pore regions with high permeability.

### 4.3 Characterization: third step

#### 4.3.1 Dynamic light scattering tests

**Fig. 4:**
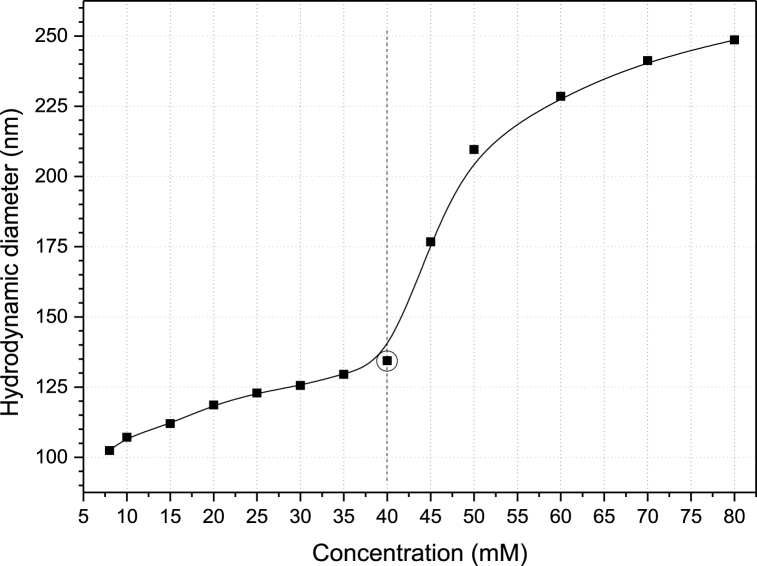
Graph showing the variation of Microemulsion droplet size as a function of SDS concentration.

The dynamic light scattering technique provides a suitable method for microemulsion characterization. Microemulsion systems consisting of 1:1 heptane/aqueous compositions are described by micelles and/or micellar agglomerates in the continuous phase, depending on the surfactant concentration. Fig. 4 shows the average hydrodynamic diameter of droplets/micelles formed within the microemulsion phase at 298 K. DLS results confirm the nanometric size of micelles/agglomerates within the microemulsion (bulk) phase. The droplet size is found to be 102.4 nm at CMC, which further increases to a more pronounced micellar structure with 118.6 nm at 20 mM. With increasing SDS concentration, the average droplet size gradually increases due to the probable stretching of micellar structures from a spherical to rod-like or ellipsoidal geometry [41,42]. The microemulsion bulk primarily comprises stable micelles with a favorable degree of elasticity and structural integrity up to 40 mM, which corresponds to an average droplet diameter of 134.4 nm. This improves the interfacial activity between oil and water phases in the presence of SDS and co-surfactant molecules. The point of transition from micelles to vesicles is marked in the figure. At concentration above 40 mM, a steep ∼33% increase in ME droplet size is observed with values in the range of 176.7–248.6 nm. This is attributed to the increased degree of molecular aggregation, which transforms micelles to ‘bigger’ vesicles or agglomerates in solution [[43], [44], [45]].

**Fig. 5:**
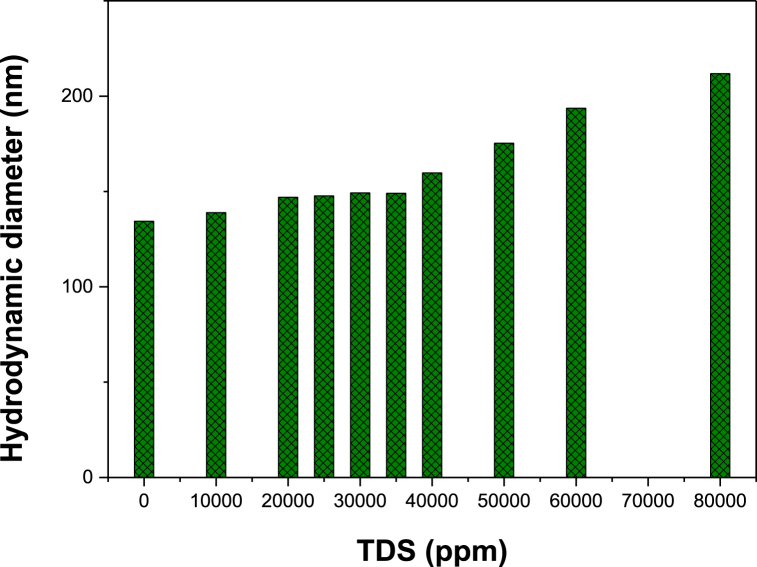
Effect of salinity on the average droplet size for ME fluid containing 40 mM, obtained from DLS measurements.

The average ME droplet size increased with increasing salt ppm TDS) content. In the absence of salt, the smallest droplet was observed due to the greater oil-water interfacial area available for SDS adsorption [46]. Salt causes extensive droplet aggregation and/or ion hydration of salts in the ME phase, which is responsible for the formation of bigger droplets [46,47]. Either of these two mechanisms results in the weakening of electrostatic repulsive forces among droplets, which leads to a coalescing effect [47,48]. The droplet size in the ME phase is found to increase slightly from 134.4 nm without salt to 138.9 nm at 10,000 ppm salt. With further salt addition, the droplet sizes gradually increase to 146.97 nm; 159.73 nm; 193.67 nm and 211.87 nm in the presence of 20,000; 40,000; 60,000 and 80,000 ppm TDS (NaCl) respectively. Fig. 5 exhibits the plot of droplet size versus salinity for analyzed {decane/SDS/cosurfactant/brine} based ME system.

#### 4.3.2 Solubilization parameters and Chun-Huh IFT

**Fig. 6:**
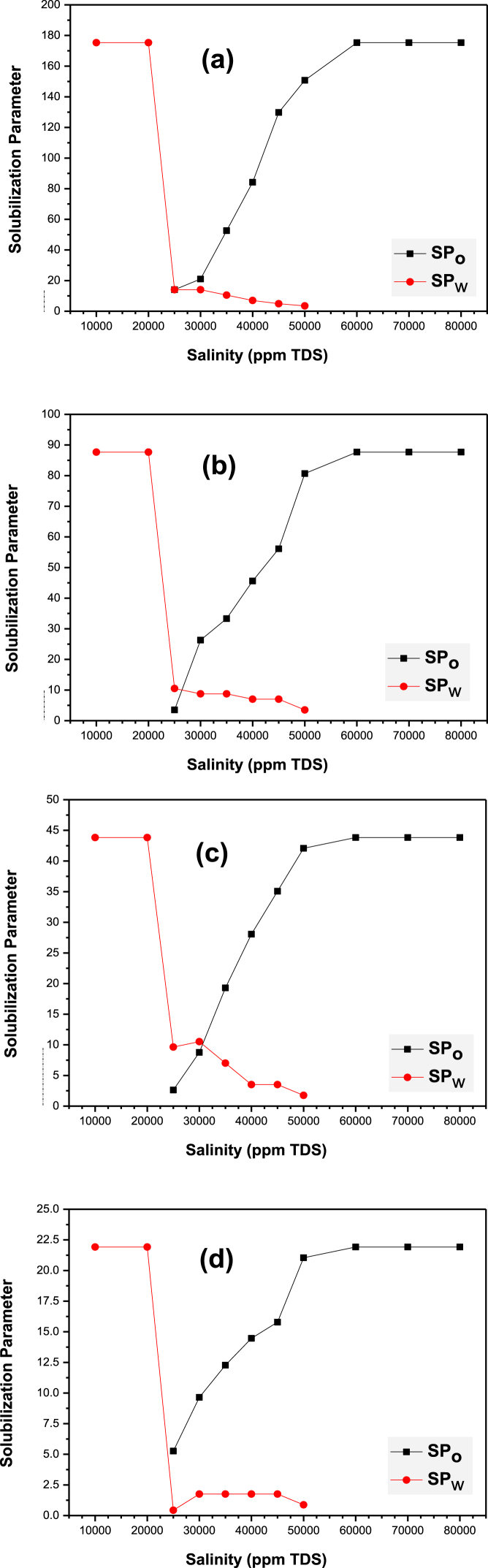
Plots showing solubilization parameters versus salinity (ppm NaCl) for {decane/SDS/isopropanol/brine} based microemulsion systems at varying SDS concentrations: **(a)** 10 mM, **(b)** 20 mM, **(c)** 40 mM and **(d)** 80 mM.

Solubilization phenomena in microemulsions is a function of the amount of surfactant available for mixing in the bicontinuous oil/water phase. In this approach, all surfactant molecules are assumed to be present in the microemulsion phase only [12]. The oil solubilization parameter (SP_o_) is observed to increase whereas the water solubilization parameter (SP_w_) decreases with increasing salinity. The degree of interactions between formation water and surfactant molecules (injected microemulsion) decreases owing to reduced hydrogen bonding and weakened hydrophilicity in the presence of salts [49,50]. This exhibits the ability of SDS molecules to block water channels in high permeability regions by decreasing their water solubilizing potential as the salinity increases. Plots showing solubilization potential versus salinity curves for {decane-SDS-isopropanol-brine} systems are depicted in Fig. 6.

At optimal salinity, microemulsions are bicontinuous, Winsor III systems which can solubilize oil as well as aqueous phases in equal proportions. Chun-Huh [51] developed an Equation (3) to calculate the IFT as shown:

(3) $$\gamma =0.3\;/\;{\left({SP}^{*}\right)}^{2}$$

where γ is the IFT and SP* is the oil/water solubilization ratio at optimal salinity conditions. The IFT values determined from the Chun-Huh correlation lie in ultra-low magnitudes of the order of 10^−3^ mN/m. The optimal solubilization and salinity condition depend on the in-situ oil/water saturation, temperature and composition. The oil and water solubilization potential curves intersect at a particular salinity for different surfactant compositions, i.e. 10, 20, 40 and 80 mM. This corresponds to the point of optimal solubilization parameter/optimal salinity condition for the prescribed microemulsion, which is characterized by equal degrees of ME hydrophilicity and hydrophobicity [52,53]. The optimum salinity conditions for SDS microemulsion systems were identified as 25,000 ppm TDS, 26,500 ppm TDS and 30,500 ppm TDS (NaCl content) for 10, 20 and 40 mM concentration respectively. No optimal condition was observed for the 80 mM SDS microemulsion system since the oil and water solubilization curves did not intersect. Table 1 depicts the optimum solubilization ratio, optimum salinity, and the Chun-Huh IFT for varying SDS concentrations in {oil/surfactant/cosurfactant/brine} systems. If the ME under analysis is employed for conditions wherein the formation water salinity is greater than the optimal salinity value, the flood-front is less likely to move in the forward direction and, hence, can effectively plug the water channels for better reservoir conformance.

**Table 1:** Optimum parameters related to solubilization and interfacial activity for {decane/SDS/isopropanol/brine} based ME systems.

SDS conc. (mM)	Optimum solubilization ratio (SP*)	Optimum salinity (ppm TDS)	Chun-Huh IFT ( × 10^−3^ mN/m)
10	14.03	25,000	1.525
20	10.26	26,500	2.850
40	10.02	30,500	2.988
80	–	–	–

#### 4.3.3 Salinity scan tests

**Fig. 7:**
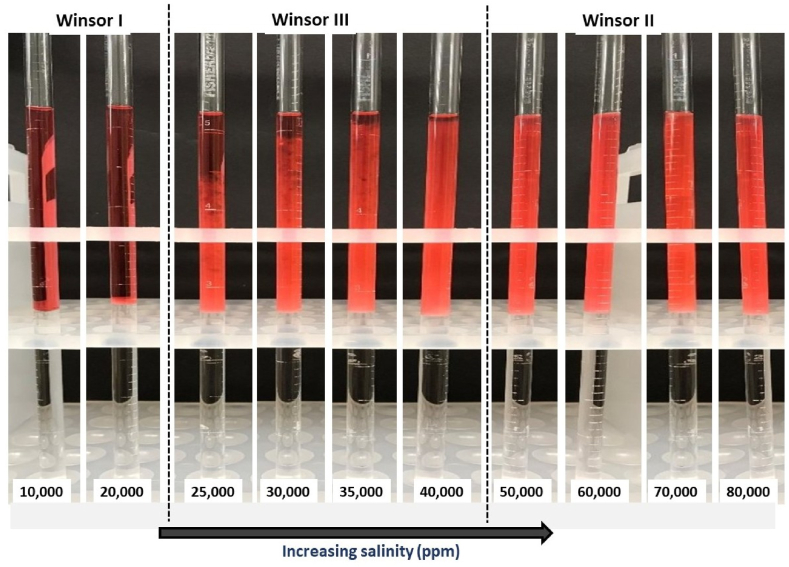
Salinity scan for microemulsion system expressed as a function of TDS (10,000–80,000 ppm) containing 40 mM SDS surfactant and 1:1 oil-to-water ratio. Results show that increasing TDS results in gradual transition of ME behavior from Winsor I to Winsor III to Winsor II.

Fig. 7 depicts the salinity scan results of microemulsion in terms of Winsor-type phases. The relative phase volumes of ME systems are observed to be influenced by salt content. At low concentrations, the Winsor I phase exists, which is described by an upper oil phase and lower microemulsion phase [54,55]. This system gradually transforms to Winsor III at 25,000 ppm NaCl, wherein the oil solubilization effect begins to increase and a three-layer (oil-microemulsion-aqueous) phase is viewed [12,56]. As salinity increases, the oil solubilization effect becomes more dominant and eventually surpasses the water solubilization potential. At ≥50,000 ppm NaCl, the oil phase is completely solubilized to form an upper microemulsion phase and the Winsor II system is observed [12,57,58].

Each of the three phases, i.e. Winsor I, II and III are considered suitable for CIT in reservoirs depending on the in-situ fluid saturation in rock pores. Winsor II is suitable in conditions where the water production needs to be blocked with fluid diversion capabilities to low-permeability zones [58]. Being water-in-oil microemulsions, they are unable to solubilize additional aqueous phases in the formation and henceforth, aid in controlling the water-to-oil production ratio. Winsor I can effectively displace and carry oil from low-permeability zones, which cannot be produced by water-flooding due to high capillary forces within pore-throats [54]. As oil-in-water type microemulsions, Winsor I microemulsions push the oil from trapped pore-throats and aid in increased oil recovery. Winsor III microemulsions are favorable for both functions, i.e. pore-plugging and oil displacement as per localized reservoir needs [4,55]. With increasing salinity, the oil-surfactant-isopropanol-brine is capable of altering ME phase behavior from an oil-in-water type (Winsor I) to a three-phase ME (Winsor III) to a water-in-oil (Winsor II) system.

### 4.4 Physicochemical evaluation: fourth step

#### 4.4.1 Dynamic viscosity behavior

**Fig. 8:**
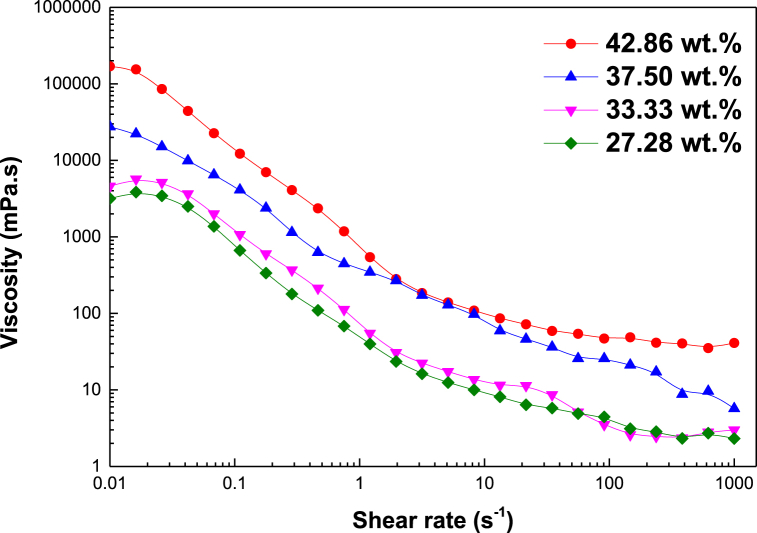
Influence of oil content (wt.%) on the viscosity versus shear rate plots for {decane/SDS/isopropanol/brine} ME system containing 40 mM SDS concentration and 1:1 cosurfactant-to-surfactant ratio.

Fig. 8 presents the effect of oil content on the viscosity versus shear rate plots for {decane/SDS/isopropanol/water} based ME fluids. As expected, pseudoplastic or shear-thinning behavior is observed in the 0.01–1000 s^−1^ range due to weakened attractive forces among ME droplets. With increasing shear rates, the microemulsion droplets further move away from one another, thereby creating an elastic physical response [25,59]. This behavior is necessary to attain injectivity through the wellbore and sufficient viscosity when ME reaches deep into the formation. ME viscosity influences the flow velocity of the displacing fluid during CIT. If the viscosity is too low, the water-plugging capability decreases [60,61]. On the contrary, too high a viscosity of MEs causes injectivity issues [60,61]. The viscosity of microemulsion is found to increase with oil content in the ME system owing to variation in micellar distribution/morphology [62,63]. The viscosity of SDS microemulsions are determined at 7.34 s^−1^, representing dynamic reservoir flow condition, as 10.21 mPa s (at 27.28% oil) and 14.19 mPa s (at 33.33% oil). As oil content increases, the oil solubilization plays a dominant role and the micellar distribution becomes more pronounced with lesser free space. Further increase in oil composition significantly weakens the electrostatic repulsion effect among ME droplets and, consequently, enhances the micellar arrangement to attain a closer packing distribution [63,64]. This leads to a significant rise in the viscosity of SDS microemulsion to 104.89 MPa s and 112.45 mPa s in the presence of 37.50% and 42.86% oil respectively at 7.34 s^−1^. Favorable viscosities are observed for studied ME samples in the desired shear rate range, which confirms their ability to avoid fluid flow to large (high permeability) pore regions, which is desirable for CIT.

#### 4.4.2 Microemulsion stability and effect of salinity

**Fig. 9:**
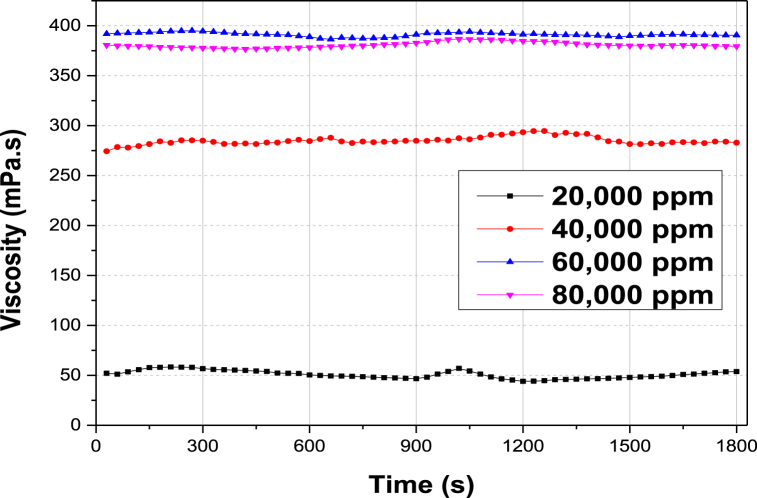
Kinetic stabilities of {decane/surfactant/isopropanol/brine} MEs containing 40 mM SDS and varying TDS content (ppm), expressed in terms of viscosity versus time plots.

The stability trait of surfactant-stabilized microemulsions for use in conformance improvement has been investigated in terms of their viscosity results. It is pertinent to note that coalescence of droplets cannot be avoided during ME formation, but the stabilization phenomenon gradually improves with time after formation [14,65]. Fig. 9 exhibits the variation of ME viscosity with time elapsed under dynamic shear conditions of the reservoir (7.34 s^−1^) at different salinities. It is observed that the viscosity of SDS microemulsions does not change significantly. The micellar structures encompassing the bulk ME phase retain their structural integrity even after 30 min of study. This occurs due to the enhanced strength of the electrostatic repulsion effect among dispersed micelles, which minimizes the coalescence effect and maintains fluid viscosity under shear [9,66,67]. Furthermore, the viscosity of ME initially increases with salt content (up to 60,000 ppm TDS) due to the improved electro-shielding effect at oil-aqueous interfaces. However, at a higher NaCl concentration (80,000 ppm), the viscosity reverses (decreases) slightly owing to the domination effect of droplet deflocculation against shear deformation [68]. ME viscosity is directly related to pore plugging in high permeability pores and subsequent fluid diversion to low permeability regions where the crude oil is trapped by capillary forces.

### 4.5 Detailed assessment of experimental results: final step

The surfactant attributes and microemulsion behavior are properly investigated for formulation design. The strategy is based in initial surfactant screening analysis in terms of cost, market availability and physical properties. Thereafter, its suitability as an emulsifier for microemulsion (ME) formation, ternary behavior and phase behavior are assessed. The favorability of ME is characterized in terms of size analysis, solubilization, and salinity scan. The physicochemical evaluation is performed for the ME fluid system(s) in terms of viscosity and stability. Other important factors for technical assessment include cost factors; the environmental footprint of the microemulsion fluid; and ease of application pertaining to reservoir needs. This is useful to make an informed decision about ME formulation strategy for application in CIT. Table 2 shows a decision table, which objectively specifies the tools, factors/parameters and requirements for an effective ME formulation strategy in the laboratory.

**Table 2:** Decision table highlighting the different aspects of ME formulation in the laboratory for technical assessment in conformance improvement.

Stepwise Methodology	ME Formulation Techniques	Outcomes	Strategy/Decision
**Surfactant screening & analysis**	**Cost analysis**	Low cost of procurement	**Proceed**
		Very high costs involved	**Abort**
	**Market Accessibility**	Readily prepared or available	**Proceed**
		Not available, or incur high transportation costs	**Alternative**
	**HLB and micellization**	Ideal HLB in the 8–12 range, low CMC	**Proceed**
		Unfavorable HLB but low CMC	**Proceed**
		Very high CMC, leading to high rock retention tendency	**Alternative**
**ME Phase behavior analysis**	**ME synthesis route**	Employ suitable method in lab	**Proceed**
	**Ternary phase diagram**	Identify favorable 2+ phase systems in the presence of low surfactant conc.	**Proceed**
		Identify favorable compositions, but with high surfactant requirement	**Alternative**
	**Phase visualization**	Winsor I, II, III types with low coalescence rate	**Proceed**
		Phase separation occurs quickly (few mins-hrs)	**Abort**
**Characterization**	**Dynamic light scattering**	ME droplets measured in nanometric range	**Observe, Analyze**
		Effect of salinity on droplet size	
	**Microscopy**	Droplet morphology and size	**Observe, Analyze**
	**Solubilization studies**	Optimal solubilization and IFT behavior	**Observe, Analyze**
		Relative tendency to solubilize water/oil	
	**Salinity scan**	Phase transition among different Winsor types	**Observe, Analyze**
		Relative Phase volume curves with salinity	
**Physicochemical evaluation**	**Viscosity**	Pseudoplastic flow character with moderate to high viscosity under shear	**Proceed**
		Pseudoplastic behavior with very low viscosity	**Alternative**
		Dilatent flow behavior	**Abort**
	**Stability**	Viscosity and/or zeta potential remains nearly constant with elapse of time	**Proceed**
		Fluctuating trends in viscosity/zeta potential with time	**Alternative**
**Economic factor**	**Cost analysis**	Functional with low/manageable operating costs	**Proceed**
		Scope for incurring significant costs during setup, maintenance and operation	**Abort**
**Environmental concern**	**Degradability**	ME components are degradable or can be retracted	**Proceed**
		Leads to rock retention and formation damage	**Abort**
		Toxic in nature, and potency to spread to nature	**Abort**
**Reservoir needs**	**Porous media flow studies**	ME shows favorable behavior in desired rock systems	**Proceed**
		Undesirable features and rock-fluid incompatibility	**Abort**

Decision columns for each outcome are marked in different colors as shown. Green = Proceed with the strategy; Yellow = Search for alternative approach; Red = Abort the formulation process; Blue = Observe and analyze the result only.

#### 4.5.1 Viscosity-stability relationship

The principle of conformance improvement applies specifically to understanding the relationship between stability and viscosity of the fluid. This can be applied to define the requisite properties of microemulsions as a promising alternative to CIT. A major factor that influences ME behavior is the emulsifier concentration. In general, the presence of emulsifier(s) renders stability and volume to the ME system. The size of ME droplets increases with increasing surfactant concentration, which allows additional SDS molecules to accommodate onto the interface. For complex fluid systems such as MEs, stability is not always inversely proportional to the micelle (droplet) size [69,70]. Factors such as coalescence rate; hydrophobic and hydrogen bonding effect; and degree of electrostatic interactions among dispersed micelles also play an interesting role in ME stability [14,70,71]. The morphology and arrangement of micelles affect the viscosity behavior of emulsions. In addition, MEs exhibit a pseudoplastic flow behavior which allows better injectivity at the surface and improved mobility control within the sub-surface pores [17,72]. The ME fluid must also exhibit sufficient viscosity, which is necessary to reduce the mobility ratio through high-permeability zones [17,73]. For conformance improvement, surfactant-stabilized MEs should exhibit adequate viscosity and pseudoplastic flow character under shear, whilst also retaining sufficient fluid integrity.

#### 4.5.2 Data analysis and parameters consideration

The reproducibility of data must be good within acceptable limits for surfactant ME fluid systems to achieve favorable conformance. To the best of our knowledge, no laboratory workflow on ME-CIT formulation methods has been reported till date. Therefore, this article aims to suggest a standard decision-making tool from scratch for ME optimization. A favorable cosurfactant-to-surfactant ratio (1:1) has been chosen in this study to avoid a large reduction in viscosity. It is necessary to analyze the same ME compositions via characterization and physicochemical evaluation tools in duplicate in order to ensure similar results for application in the industry. Also, the reservoir conditions such as salinity must be considered before confirming its suitability for conformance [74,75]. For instance, the SDS microemulsions discussed herein showed good results up to salinity level of 80,000 ppm TDS, which marks its limit for a favorable CIT process. Microemulsion fluid consisting of 40 mM sodium dodecyl sulfate and the cosurfactant-to-surfactant ratio of 1:1 showed very favorable results in terms of droplet characteristic, stability and viscosity; and warrants utility as a conformance fluid. This was further validated with the aid of flow control experiments by injecting the ME fluid, post water-flooding in a simulated laboratory reservoir model [76]. Investigations revealed a sharp pressure drop with a favorable reduction in water-cut percentages (as low as 30%) during optimized ME flooding, which is attributed to the effective plugging of water channels within the high permeability zones of the rock.

The technical analysis obtained from experiments must consider cost, environment and market availability factors. The microemulsion must comprise low to moderate cost components, which reduces the operational costs of the industry [4,77]. The use of non-degradable surfactant/co-surfactant materials with high rock retention ability must be minimized for beneficial ME formation [12,78]. The emulsifier compounds (surfactant, cosurfactant, particles, etc.) must be readily available or a commercial setup must be already in place [29,77,78]. In addition, the ME fluid must be non-toxic to humans, animals, and the natural environment, per corporate social responsibility (CSR) of the industry.

## 5 Conclusions

The study develops a comprehensive laboratory workflow to identify beneficial formulations of surfactant-based microemulsions for conformance improvement. The application of the proposed approach based on anionic surfactant-based ME systems provided an interesting perception from a research viewpoint. The study is restricted to the selection and utilization of a specific injection (displacing) fluid for conformance control. The approach requires a full-scale setup in the laboratory to give an idea of the suitability of microemulsion in the laboratory. However, upscaling studies further need to be designed to use the laboratory results in real-field projects. The experimental case study was delineated to serve as a robust guideline for future application. The key findings are drawn as follows.

• A microemulsion (ME) formulation consisting of sodium dodecyl sulfate (SDS) exhibited potential for conformance improvement. The system exhibited good results upto 80,000 ppm TDS as a measure of salinity.
• The workflow methodology for ME formulation has been generalized into five steps: screening, phase behavior analysis, characterization, physicochemical evaluation, and technical assessment. The approach can be employed to optimize any surfactant system into a ME-CIT tool with the scope for flow-displacement testing.
• It is necessary to categorize ME compositions based on Winsor type. This is followed by characterization tools in the laboratory, which confirms the validity of the system in terms of size, morphology and solubilization potential.
• The reservoir conformance ability of ME fluid is measured in terms of two important physicochemical traits: stability and viscosity.
• The technical assessment involves the combined expertise of both research and industry. Besides result analysis, it should also encompass the cost-effectiveness of the process, chemicals’ market availability, reservoir needs, and environmental concerns.

## Author contribution statement

Nilanjan Pal: Conceived and designed the experiments; Performed the experiments; Analyzed and interpreted the data; Contributed reagents, materials, analysis tools or data; Wrote the paper.

Yara Alzahid: Analyzed and interpreted the data; Contributed reagents, materials, analysis tools or data.

Abdulkareem M. AlSofi, Muhammad Ali, Nurudeen Yekeen: Contributed reagents, materials, analysis tools or data.

Hussein Hoteit: Conceived and designed the experiments; Analyzed and interpreted the data; Contributed reagents, materials, analysis tools or data; Wrote the paper.

## Data availability statement

Data will be made available on request.

## Declaration of competing interest

The authors declare that they have no known competing financial interests or personal relationships that could have appeared to influence the work reported in this paper.
